# Living normally without being oneself: A qualitative study on the experience of living with advanced chronic kidney disease

**DOI:** 10.1371/journal.pone.0295506

**Published:** 2023-12-21

**Authors:** Carmen de la Cuesta-Benjumea, Luis Eduardo Hernádez-Ibarra, Claudia P. Arredondo-González

**Affiliations:** 1 Department of Health Psychology, University of Alicante, Alicante, Spain; 2 Faculty of Nursing and Nutrition, Autonomous University of San Luis Potosí, San Luis Potosí, Mexico; 3 Department of Health Sciences, San Juan de Dios Foundation, Pontifical University of Comillas, Madrid, Spain; King Khalid University, SAUDI ARABIA

## Abstract

The aim of the study was to describe and analyze the experience of people with advanced chronic kidney disease. Chronic kidney disease is a growing public health problem that is on the increase worldwideThe experience of living with this illness is paradoxical, as it can include feelings of dependent autonomy, distant connection, abnormal normalcy, and uncertain hope. Every chronic disease involves a biographical alteration from the onset. For those who suffer it, it implies a breaking down and reconstruction of their everyday life. Despite the prevalence of the disease and the increase in the number of qualitative research studies in recent decades, there has not been much research on the experience of people with Chronic kidney disease. This is a qualitative study that began in 2018 and concluded in 2021.Twenty-one people with advanced chronic kidney disease were interviewed. They participated voluntarily with informed consent Participants were selected by purposive sampling. Data analysis was guided by grounded theory procedures using the Nvivo 12 software. This study reveals that people with advanced chronic kidney disease do not feel the same as they used to because their control over their lives is limited; because they feel their health is in a continuous state of deterioration; and because of the changes in themselves and in their relationships with others. With chronic kidney disease, their identity is continually called into question. The normal lives of these people–their biographical constructions–are precarious and are continuously being remodelled by the effects of treatment and the inexorable course of this disease. This study contributes to an understanding of the experience of people with advanced chronic kidney disease. It can contribute to helping health care professionals effectively support these patients in their efforts to lead a normal life and in making decisions about their treatment.

## Introduction

Chronic kidney disease (CKD) is considered a public health problem [1] that is on the increase worldwide [2, 3]. Cardiovascular risk, together with the terminal stage of the disease, significantly affects the burden of mortality and morbidity around the world [1, 4]. In 2017 it had a prevalence of 9.1% (697.5 million cases), and it is estimated that just over 3 million people are in the terminal stage [5]. Almost a third of all cases were reported from China and India [4]. Multiple calls have therefore been made for countries to develop strategic national action plans for the prevention and early detection of CKD [6].

The course of CKD is progressive. The most advanced manifestation of the disease is end-stage chronic kidney failure, and it requires renal replacement therapy by dialysis or kidney transplantation [7]. This treatment can lead to a repeating cycle between dialysis and kidney transplantation [8].

It is estimated that only 2% of all people with CKD reach the stage of replacement therapy; most of them die first, mainly due to cardiovascular incidents [1]. In 2005, some 40,000 people in Spain were in replacement therapy, and this number is expected to increase due to aging population and other chronic processes [9]. Renal replacement therapy consumes 5% of health budgets, making it one of the most expensive chronic disease treatments [10]. CKD is classified as a disease of catastrophic expenditure [11] and therefore, a generator of health inequalities. In 2010, it was estimated that at least 2.28 million people may have died due to lack of access to treatment for CKD, mainly in Asia, Africa and Latin America [12].

People with CKD perceive that they have a poorer quality of life than the general population, even if they have not experienced kidney failure [13]. Regardless of the stage of the disease, its symptomatology greatly affects the quality of life of people with CKD. Moreover, as kidney function deteriorates, so does quality of life [14]. Having to visit the hospital three times a week for hemodialysis; having to change peritoneal fluid daily; being subject to significant dietary, social and leisure restrictions and the loss of independence significantly reduce people’s quality of life [15, 16]. Therefore, when choosing a therapy, one of the main considerations of people in the final stages of CKD is maintaining the highest possible quality of life [17, 18].

More and more studies carried out in the area of health explore, from different approaches, the experiences of those who suffer from a chronic condition. A clear example is Good [19], who acknowledges the body of those who suffer from a chronic condition as a creative source of experiences, which are in turn determined and shaped by the context. Castro and Farina [20] point out that the body is not an abstract, mute, measurable object, but rather that it feels sensations and is reconstructed through perception, action and thought.

A systematic review described the experience of living with CKD as paradoxical, as it can bring about feelings of dependent autonomy, distant connection, abnormal normalcy, and uncertain hope [21]. Fatigue and uncertainty are two experiences that people with CKD suffer in the final stages [22, 23]. In addition, people with end-stage CKD live with a significant burden of multiple and concurrent symptoms, which impact their lives in various ways. The study participants reported up to 22 different symptoms and reported their physical decline, reduced participation in social life, and dietary restrictions [24].

Every chronic disease involves, from its onset, a biographical alteration. For those who suffer it, it means the destruction and reconstruction of their everyday life. Chronic diseases require continuous adaptation to rebuild a life in which one can live in such a way that life feels natural again [25]. When the disease interferes with people’s aspirations, the changes that take place during the course of the disease force continuous reconstructions of the self [26]. For this reason, rebuilding one’s sense of identity is one of the problems faced by people with chronic conditions [27].

The importance of reconstructing one’s biography when suffering from a chronic disease as well as the disease process has been widely documented in recent decades, highlighting peoples’ ability to carry out this reconstruction and their need to give continuity to their biography [26, 28–30]. It has been found that kidney failure and medical interventions in CKD alter the consistency of patients’ daily reality, so that they seek to create meaning and restore cohesion in their lives [31].

Normalization is how people with chronic diseases find ways to live their lives despite their symptoms [32]. “The struggle for a normal life” is an essential category for understanding the experience of people with chronic diseases [30, 33]. This refers to the efforts made by people with chronic processes to understand the changes in their lives, to integrate these changes into their lives, to seek answers to the questions posed by the disease, and to alleviate their suffering [33].

Chronic diseases often lead to drastic reductions in energy, strength, time, money, and relationships. In the case of CKD, a decrease in physical function and social relationships, work restrictions, diet, and loss of independence have been documented [15, 34]. Peritoneal dialysis patients report how the inconvenience disrupts their daily activities due to the peritoneal catheter. Some effects include longer showers, having to replace the dressing immediately to prevent infection and being limited in their clothing options [17]. In order to overcome these restrictions and inconveniences, patients carry out strategies that they feel add to their workload [27]. A systematic review confirms that adults with end-stage CKD do difficult, time-consuming, invasive, strenuous tasks that impact all aspects of their lives [5].

Despite the prevalence of the disease and the increase in qualitative studies in recent decades, there has not been much research on the experience of people with CKD [35]. Living with a serious chronic disease requires support from health professionals whom patients can trust, so it is important to understand the experience of people with high-impact chronic diseases in greater depth [33]. Hence, the aim of this study was to describe and analyze the experience of people with advanced CKD. The results contribute to an understanding of the illness experience of these patients.

## Materials and methods

This is a qualitative study that employed grounded theory procedures [32, 36, 37] to analyze the data. Grounded theory takes the symbolic interactionism perspective where people are considered to be involved in a continuous process of problem solving and sees meanings as derived and transformed through people’s interactions [38]. Symbolic interactionism stresses people’s agency in the social world and emphasizes the importance of the conditions under which their interactions take place.

### Ethics statement

The study was approved by the ethics committee of the University of Alicante (UA-2018-12-02). It began in November 2018 and concluded in June 2021.

Potential participants were contacted through patient associations and the staff of a hospital hemodialysis unit. They were fully informed and given the opportunity to discuss the study face to face with researchers. Participants signed their consent to participate. Their right to withdraw from the study at any time was respected. During interviews participants emotional distress was assessed and provided the required support. To ensure privacy, data was anonymized and stored in encrypted devices and locked cabinets.

Twenty-one people with advanced CKD participated voluntarily in the study. Participants were selected by purposive sampling and They were men and women who had had chronic kidney disease for 2 to 51 years and who were undergoing hemodialysis or kidney transplantation. Their ages ranged from 30 to 81 years, with an average age of 54 years. More than half of the participants completed secondary school and some of them engaged in further education, only one participant did not have studies. All participants were treated within the national health service, and most were retired due to their illness (see characteristics in Table 1).

**Table 1:** Study participants characteristics.

Participant’s code	Sex	Age (years)	Years with CKD	Treatment	Education	Employment status	Health Insurance
E2	Male	49	25	RT	Secondary school	Employed	Social Security
E3	Male	53	51	RT (reject) HD	Vocational training	Retired due to illness	Social Security
E4	Female	52	6	RT	Secondary school	Retired due to illness	Social Security
E5	Female	37	22	RT	Vocational training	Employed	Social Security
E6	Female	46	20	RT	Primary school	Retired due to illness	Social Security
E7	Male	37	37	RT	Secondary school	Employed	Social Security
E9	Male	55	8	RT	Vocational training	Retired due to illness	Social Security
E10	Female	38	10	HD	Vocational training	Employed	Social Security
E11	Male	30	28	RT (reject) HD	Vocational training	Retired due to illness	Social Security
E12	Female	49	18	HD	Secondary school	Homemaker	Social Security
E13	Male	72	6	HD	Primary school	Retired	Social Security
E14	Female	72	2	HD	Primary school	Retired due to illness	Social Security
E15	Male	50	3	HD	Vocational training	Retired due to illness	Social Security
E17	Female	76	5	HD	Primary school	Retired due to illness	Social Security
E18	Male	68	12	HD	University	Retired due to illness	Social Security
E19	Male	58	6	HD	Vocational training	Retired due to illness	Social Security
E20	Female	81	20	HD	None	Retired due to illness	Social Security
E21	Female	66	35	RT	Secondary school	Retired due to illness	Social Security
E22	Male	57	2	HD	Primary school	Retired due to illness	Social Security
E23	Female	46	7	HD	Secondary school	Retired due to illness	Social Security
E24	Male	55	36	RT	Vocational training	Retired due to illness	Social Security

### Data collection and analysis

A total of 21 semi-structured interviews were carried out in the participants’ homes, in the hemodialysis unit, or at the patients’ association. The interviews lasted from one to two hours. They were recorded and fully transcribed. The interviews were individual, except for five interviews in which a family member was also present (see S1 File). Data collection evolved over time; interviews were analyzed consecutively to inform the next interview.

Data analysis was guided by grounded theory procedures and developed in three main stages. First, once data was entered into the Nvivo 12 software, open coding was carried out, from which the study categories emerged. Second, categories were developed through focused coding and the constant comparison strategy. Thirdly, emerging categories were refined, compared to the data for fit, and were saturated. As the analysis progressed, successive memos with different levels of abstraction were written. Initially they were descriptive and focused on codes, later became conceptual describing emerging categories. In the final stage of analysis memos were grouped according to the main categories and integrated into a core memo that identified and described the central category with its subcategories [36, 37]. Grounded theory procedures were completed concurrently in a continuous growing process of data conceptualization.

### Rigor

The rigor of the study was observed by facilitating the emergence of data by means of open questions asked in the interviews. Emergence of the categories was ensured through inductive analysis and by the constant comparison strategy. Throughout the analysis process, the categories were compared to the data for its fit. A reflective journal was maintained during the fieldwork, and reflective notes were made during the data analysis.

A core category emerged from the analysis, made up of two subcategories that portray the experience of living with advanced CKD. One of the subcategories describes the context of this experience and the other the strategies that people with chronic kidney disease use to normalize their lives.

## Results

This study reveals that the central phenomenon of the experience of living with CKD is that of “*living normally without being oneself*.” The phenomenon is described through the subcategory “not being the same person one used to be,” which reveals the context of this experience, and the subcategory “leading a normal life,” which tells of the means that people with chronic kidney disease must employ to deal with everyday life. Each of the subcategories are presented below.

To preserve the anonymity of participants, codes are used in the text below and any names associated with the data have been changed to fictious names.

### Not being the same person one used to be: Context of living with chronic kidney disease

During the interviews, the study participants stated that they were no longer the same person they used to be. By this, they were referring to the physical limitations and the complex and interconnected psychosocial processes that result from the treatment and the disease. One participant who had been a sportsman put it succinctly:

“…you don’t feel like playing, because what you want is to be yourself again, like I’m not who I used to be.” (E11)

The feeling of “not being the same person one used to be” accompanies people with CKD throughout the course of the disease. Their life, their plans, their body, and their self are permanently and continually transformed, so they are not the same person in different ways. Thus, data analysis showed that the context of the experience of people with CKD is made up of three interrelated conditions: 1- Lacking freedom and being on hold; 2- progressive impairment; and 3- the dissonances in who one is.

#### Lacking freedom and being on hold

The participants go from feeling that they had a life of liberty to a life of obligations and restrictions. They must follow a strict diet and adhere to treatment schedules that govern a large part of their lives. Thus, for example, there are participants who feel “enslaved” and tied to a machine (E23), lacking the freedom they used to have to travel or socialize (E3; E4; E17; E19). Their multiple therapeutic obligations and restrictions can make their lives ‘a misery’ (E4). A participant talks about the restrictions they must follow:

“The hardest… I think the most difficult of all is not drinking; to monitor your drinking and eating; the number of things you must do to cook three meals, wash the food twice to remove the potassium; fruit, we can’t eat it, or not too much; cheese neither, there are a lot of things, the diet is very strict.” (E23)

Nevertheless, treatment opens a window of temporary freedom in people’s daily lives. When the patient is waiting for a transplant, the period is uncertain but hopeful. About this hopeful uncertainty, one participant said:

“… well, I already know that when they take this thing [dialysis] away, it will be high time, although you always have hope: ‘So maybe they’ll give me a transplant,’ but in the past it took a long time, and they never tell you. ‘It could be now,’ they tell you; it could be now, or it could be in fifteen years.” (E14)

Thus, there is the wait for a transplant with the expectation of improvement, of being able to lead a “normal life” (E11; E15); waiting for a technological advance that will make it possible, such as a “bionic kidney” to eliminate obstacles to the transplant or incompatibilities; or advances in stem cell research (E4; E5; E11; E15). They wait for each dialysis session with the expectation of being able to get to the following session until they receive the call that a compatible kidney is available. In an interview a participant talks about this hopeful wait:

“You know that you’re on the waiting list and that sooner or later they will call you. Well, all of us on that list have that hope.” (E21)

The wait can last up to ten years (E3) and is fraught with uncertainty. Participants do not know how long they will be in treatment for, be it dialysis or immunosuppression; whether they will be able to self-manage it (E4); whether they will recover after a kidney transplant or will they experience rejection; and if everything goes well, how long will the transplanted kidney last for, because as one participant said, “back to dialysis you go” (E 3) and with it the patient returns to the cycle of hopeful and uncertain waiting, and conditional freedom.

Under these conditions of waiting and absence of freedom, the study participants feel that they have lost control of their lives.

#### Progressive impairment

Treatment halts the fatal course of the disease, but it is not without side effects; it has increasingly high health costs for people with CKD. The study participants commented that dialysis was fatiguing and wore them down (E12; E14). It is a physical and psychological exhaustion (E21) that over time they get worse as one participant says:

“I changed for the better when I started with this, I changed for the better. But of course, it’s been so long, now it’s changing for the worse! Always worse, if it hadn’t been for that (points to the machine), I wouldn’t have been around since more than a year ago. It changes you.” (E20)

People who receive transplants are vulnerable to infections because they are immunosuppressed (E3; E4; E7; E9). These infections can be very serious, as was the case of E5, who had a cytomegalovirus infection. In addition, they may have complications such as bleeding, kidney stones or repeated urinary infections (E4; E5), and they may even reject the transplanted organ (E2; E3; E6). The deterioration in their condition is exacerbated when a patient has several transplants and must go back to dialysis after each rejection, something that is not uncommon in the long course of living with CKD. In addition, the disease does not stop completely with treatment and its progress leaves sequels (E2). One participant talks about how he sees his diminished capacity:

“…you get tired right away, when your kidneys are bad you can’t say, ‘well I’m going to do this,’ because you get tired, you get exhausted, you can’t keep up.” (E19)

As time goes by, people feel that they are becoming older and sicker (E20); that they don’t have the strength to cope with daily living, as one participant eloquently comments:

“…we invalids don’t have a life anymore. Those of us who are younger still seem to have a little more strength, but as time goes by, it goes to shit, to put it bluntly. Your bones turn to dust, you have fatigue, fatigue most of all, energy, I used to be very energetic, very active and this disease has left me a f----nobody, pardon my language, no strength, energy, nothing, psychologically.” (E23)

The study participants feel that they are no longer the same physically because the treatment and the disease leave increasingly deeper imprints on their health and, as we will see below, on their self.

#### The dissonances in who one is

Appearance, personality, and relationships with other people are also altered by the disease and its treatment. The accounts of the participants are full of references to changes in appearance such as this comment by a participant:

“Because, for example, now I have this big belly and I wonder, ‘how long am I going to have this?’ Because you know, this belly, I always weighed 50 kilos, 55, 56, now I weigh 66, ten kilos more than I used to, and this deformity, I’ve never had this deformity before in my whole life.” (E4)

Their personality and mood are altered; they feel that they are not the same, that they have lost a sense of security in themselves and in what they can achieve (E23). They experience mood changes; highs and lows (E3). “Down” times alternate with times of hope for improvement (E15), and this affects their relationships:

“Yes, it weighs on your personality too, your mood, since you’re not well, you want to do things and you can’t and all that, because your mood, your character … since I’ve been sick, my personality has got worse; all you can do is endure, I have more bad moods, I get upset easier, I don’t know, I talk back more without meaning to, but it’s because of my illness.” (E23)

Moreover, work, and social life are disturbed by chronic kidney disease and its treatment, and with it the sense of who one is. They may have to give up being in contact with friends (E15); some must look for new jobs (E2–E12); others find it very hard to do their jobs (E21–E5) and others simply cannot continue working (E15). When they start dialysis, they are classified for workplace purposes as having developed an “body part disability” (E24). About these changes, one participant says:

“I used to be a very active person. I would leave at eight in the morning and come home at one, I would go back to work at three and get home at nine at night. In other words, I had a very active working life.” (E6)

The disease and its treatment might disturb an essential part of who one feels he/she is. Its significance is that it not only alters the present but also disrupts persons’ future. One study participant gives an example of plans cut short by her illness:

“This disease really does have serious consequences; you say, I am limited now, I can’t have children anymore and maybe I wouldn’t have had more anyway, but it has taken away the possibility. You start thinking it’s crazy, but that’s how it is. I mean, I’ve always wanted more. I don’t know, maybe if I’d been healthy, I would have had more. But I always wanted to. I always did.” (E6)

The illness causes technology to invade people’s daily lives, transforming the home environment and affecting the whole family by the intrusion of the dialysis machine and equipment (E4); a constant reminder that the person is not the same as before. Under these conditions, study participants feel that they have lost control of who they are.

To sum, CKD and its treatment alter the normality of life for those who suffer from it; it disrupts their sense of who they are and what they can do. For the study participants, nothing is the way it used to be, nor are they who they used to be. The disease has turned their lives upside down. Nevertheless, as it is shown as follows, they continue to struggle to lead a life worth living, working to make the physical life provided to them by biomedical treatment compatible with social, family, emotional and work life.

### Leading a normal life: A work of biographical continuity

Although the disease breaks into and changes people’s lives, far from accepting it and resigning themselves to the conditions and limitations imposed on them by the disease and its treatment, patients work hard to have a biographical continuity; that is, to live as they used to; what the participants in the interviews called “leading a normal life.” Thus, for the participants in the study life is not lived whatever the cost but rather as a life that is worth living in a specific way; that is, in such a way that the restrictions imposed by the disease and treatment do not empty their lives of meaning. One participant expresses it eloquently:

“…so, the way things are, of course you say what do I want to live for if I can’t lead a more or less normal life?” (E12)

Indeed, living normally is the study participants’ main concern. This is so important for them that when choosing one type of treatment over another, it may be the main criterion: to choose the treatment that in their view interferes the least with their lives:

“The kidney must be compatible … and there is always the danger of rejection, and then the medication you have to take to be able to live with it. You have to take it for life; I’d rather have dialysis and that’s it.” (E18)

What is at stake is mitigating the biographical disruption entailed by CKD. This means making an effort, as one participant explains:

“… I lead a normal life, but of course for me to do so, to have the same life as my coworkers, it could be that I need to make more effort, or it makes me more tired.” (E2)

The effort to lead a normal life can be so exhausting and demotivating that they have to give up some bits of normalcy. During the study, one participant told how his desire to travel has been killed by all the preparations that he would have to make. (E23) In addition, he would have to make this effort under a state of progressive impairment of his physical and mental health. Normal life, understood as the life they had before the disease, is thus actually a longing or a dream, something that both motivates and frustrates them, as a participant explains:

“Because if you want to go away, somewhere other than where you live, then you have to be looking for the, what do they call them, the dialysis sites, so that they can dialyze you. If there’s no room or there are no facilities, well, you stay home and don’t go anywhere.” (E17)

#### Living a normal life: The means patients employ to deal with everyday life

Living normally becomes, therefore, a life of labor, made up of adjusting and giving things up, intense management of symptoms and a search for solutions to the problems entailed by the disease and its treatment. The data analysis shows that to achieve a normal life, people with CKD carry out three strategies: 1- living within their limits, 2- protective governing, 3- remedying deficiencies in the system.

### Living within their limits

“… you must keep on living your life like you used to as far as possible, or just doing what you have to do. Whatever your condition allows.” (E9)

The study participants live within the limits imposed by the disease and its treatment, striking a balance and adapting their lives. They do everything possible to reconcile the life of a healthy person with the treatment of a sick person. For example, they organize their schedules to attend medical appointments without missing work (E2; E24); they are dialyzed at their workplace (E2); they look for the best working hours and shifts so that treatment interferes as little as possible with their home life (E4; E21) and work (E7; E12); to eliminate fluids, they substitute the sauna for exercising (E3); they overcome barriers to be able to continue traveling as they did before (E24); and they design strategies to maintain their social relationships. As one participant explained:

“The thing is, if I know that I’m going out with friends, for example, on a Saturday night, for example, then on Friday, Saturday morning I work out at the gym, and I know that at night I can drink a coke, well, not a coke, a bottle of water, which for me is a lot, darn it, it changes everything (…) other people have to know too, when you interact with people.” (E3)

They adapt to their physical limitations, doing things more slowly and taking breaks (E15); they slow down their pace at work (E2). They adapt to the treatment, seeing it as a routine (E3) or as a job they must do regularly (E21). Lastly, they adapt their social life to be able to continue it:

“No, we haven’t stopped doing anything, the only thing is that we used to go to town more, but not now, now we go less often but we haven’t actually stopped doing anything.” (E14)

When study participants live within their limits, it enables them to give their lives biographical continuity.

#### Protective governing

The study participants govern their lives to carry out a regime that allows them to stop the disease from preventing them leading a normal life, from throwing their life off balance and affecting those close to them. They protect themselves “by being very careful,” as one participant put it (E2). For example, they are careful when preparing meals (E23), they create daily exercise routines, adjust their medication to control their blood pressure (E6), measure the amount of fluids they can drink according to the dialysis treatment cycle (E23) and use tips and hacks to be able to comply with their treatment in non-standard conditions:

“So, imagine in summer! it’s so hot and you can only drink a half liter. [E: What do you do?] Well, I eat lemonade popsicles, I make lemon ice cubes and they refresh me or quench my thirst and the lemon peel also satisfies my thirst a little, you must have these little tricks.” (E23)

They also protect themselves emotionally; for example, some avoid making friends during hemodialysis because of what may happen to them (E3), and they avoid thinking about their situation:

“I don’t want to think about it more than I have to, I’m here because I’m here, that’s it, how long will it last? As long as it does, and when it’s over, it’s over! On a practical level, I don’t want to, because sometimes things change, and I just don’t want to think about it. If I have two years left or twenty-two, I’ll live them. For now, when I get out of here [hemodialysis] I’m going home, like, I’ll go to sleep, and I don’t want to think too much about it.” (E19)

They also protect their relatives by not scaring them with the news that their disease has worsened. They try to find the best time to give the news (E4), or they refuse to have compatibility tests done:

“Because my sister, and everyone, wanted to get tested to see who could give me a kidney. But it was like, burdening them for life with one kidney, and I kept thinking: ‘anything happens to them, their one kidney stops working and they don’t have the other one any more,’ it was like saying, ‘well, I’m ill myself, but I don’t want them to be ill too’… so I told them, ‘no, I don’t want any of you to do the tests, when I get the transplant I get it and there it is, but no, I don’t want to, I don’t want to permanently affect your lives’.” (E6)

Protective governance maintains the biographical continuity forged by study participants by living within their limits, making their lives as close to what they used to be as their condition allows them.

#### Remedying deficiencies in the system

While people with CKD acknowledge that the biomedical treatments, they receive are effective, they wish they had support in the day-to-day management of their disease. In an interview, a respondent says that the precarious situation in which they find themselves relates to more than just biomedical needs:

“Today at XXX [name] city hall they don’t know where to send us, because we go to the national health service offices and they tell us, ‘No, no, you have to apply for that at the social services office’ and we go to social services and they say, ‘No, no, you have to apply for that at the health service office.’ Why? Because we are organically disabled, and they still don’t have a clear understanding of what organic disability is, or which group to put us in’.” (E24)

During the interviews, the needs mentioned most by participants were for psychological support, nutritional information, and information on social benefits and how to navigate the social security bureaucracy (E3; E4; E6). They resolve the deficiencies they encounter by searching for information they lack, by contracting services outside the public health system, and by joining together in support associations (E2; E4; E6). Many of them can get problems solved through the association:

“So, in XX [name of the association], Pilar told me, ‘You know we have Javier in the psychology office and so on,’ and I said, ‘yes, thank you,’ and a few days later I said to my husband, ‘Honey, I called Javier and I made an appointment with him,’ and he said, ‘great, whatever you need.’ And oh Lord, I’ve been seeing him for a year, and he has helped me so much. Really a lot.” (E6)

By facing and dealing with the shortcomings of the system, study participants gain the necessary support to give continuity to their biographies.

#### “Normal life”

The study participants normalize their lives by means of the strategies described in the previous sections. The normality they achieve is not stable; rather, it is precarious. It could be said that they build different normalcies in response to the disease and its treatment. As the disease progresses and therapeutic options become more aggressive, life becomes more restrictive. The study participants speak of leading a “semi-normal” life (E7), or of living “more or less within normal parameters” (E6), or of “going on living” (E23). Normal life is a longed-for dream, as expressed by someone who has received a transplant:

“We hope it will last our whole life [the transplanted organ] so that we can all lead a normal life, but you continue with your limitations and stuff if you want to take care of a grafted organ that isn’t yours. With your medications and things … you can’t stop taking the medicines and sometimes they have other side effects.” (E21)

When the limitations are such that they prevent a normal life, patients resort to treatment that accommodates their limitations, even if it entails a loss. Thus, they move between different versions of a normal life as illustrated by one participant:

“I was the one who asked to go back to dialysis, because I couldn’t take it anymore. Because with my condition we had reached a limit, I was in active rejection, I began to swell up, the kidney felt heavy, and I ballooned up by 22 kilos. I mean, I couldn’t even walk. My ankles, and pardon the word, my testicles were so swollen that I couldn’t lie down. It got to the point that I made appointments with the nephrologist, I told him, ‘Listen, send me to dialysis, I can’t take it anymore’.” (E3)

Normal life is being able to keep on being who you are, and this implies a great effort. People with CKD carry out this task in adverse conditions. In this adversity they may find good fortune on their side and things in their favor. In the reports of the interviews, reference was constantly made to the good fortune they had from a treatment or in general during the disease (E2; E3; E6; E11; E19). In general, they felt fortunate to be able to continue living in such difficult circumstances, making such an effort, and giving up so much:

“Yes, another day of battle, but still, we’re lucky, we are alive, we are here, I’m lucky.” (E3)

The work they do to give continuity to their life is rewarded by good fortune, which they feel is on their side. When it fails them, they try again. (E3) This normal life they build is therefore a life full of constant struggles and a certain amount of good fortune.

## Discussion

This study reveals that people with advanced CKD do not feel the same as they used to because their control over their lives is limited; because they feel their health is in a continuous state of deterioration; and because of the changes in themselves and in their relationships with others. CKD treatments affect the body, causing changes to one’s identity [39]. Given the cyclical nature of the disease and its treatment [8], it could be said that people with CKD go through repeated biographical breaks [40].

Chronic diseases change the body and the notion that a person has of themselves [25]. These two processes are interconnected, since the image we have of ourselves is related to the image we have of our body [27]. Goffman [41] distinguished the virtual identity that is attributed to appearances and the actual identity that is based on what one can do. This study shows that in CKD, both are affected to the point that people feel they are no longer the same person they used to be, and that this is a recurring sensation.

Kidney failure can send people into a negative spiral where functional capacity decreases and fatigue increases [42, 43]. Previous studies have reported that people with CKD see themselves differently from people who have other chronic conditions [31, 44]. It has been pointed out that the life of the dialysis patient is a life of waiting [31], in which one needs hope [35]. In this study we add that this hope is tinged with uncertainty. We also add a nuance related to waiting in the hope implicit in making decisions about kidney replacement therapy [18].

It has been described that uncertainty is tinged with fatalism in the case of diabetic kidney disease [45]. Here, as in some previous studies [22], we add nuance to this hope by contributing to an understanding of the various faces of uncertainty in chronic diseases. Fatigue and uncertainty are present and have been documented in chronic diseases such as AIDS, coronary heart disease and cancer in addition to CKD [46–48]. Here we show them as conditions that affect one’s sense of who one is [49].

Defining a situation as temporary prevents people with chronic conditions from having to totally redefine their lives and themselves [25]. The hope for improvement expressed by the study participants is the hope of retaining parts of themselves, or of not completely losing their sense of who they are. With situations such as the loss of autonomy, these losses are not replaced by positive views of one’s own self [50]. The loss of freedom that the study participants spoke of is one of loss of autonomy, in which not feeling like they used to, implies feeling *less* than they used to be. In addition, the circumstance of continuous progress of the disease expressed by the study respondents suggests a paradoxical struggle between not completely losing one’s sense of who one is, while holding out in the hope for improvement, and confirming a deterioration in health, and therefore in actual identity, which is built from what one is able to do [41].

The process of losing one’s sense of self that is characteristic of chronic diseases is accompanied by the efforts of those who suffer from the disease to reconstruct their sense of self [25, 30, 49]. This study shows that people with advanced CKD are in a process of continuously repairing the biographical break caused by the disease and its treatment.

Indeed, because this task of dealing with the disease is compulsory and is carried out systematically, it can become a job in itself [26]. Together with previous studies [51], the present study documents the value of this work done by patients whose objective is to live as normally as possible. It identifies three strategies carried out by people with advanced CKD to keep up the continuity of their lives: 1- live within their limits, adapting and balancing the needs caused by the disease with their lives; 2- protect themselves and others; and 3- resolve deficiencies in the system. We have found that by adapting and by retaining patches of normality in their lives, they help give continuity to their sense of who they are.

Adaptation is one of the strategies that enables them to live with the disease and not just live for the disease [30]. People with CKD employ strategies to adapt to peritoneal dialysis, such as positive self-regulation, adjustments to reduce the impact of treatment on their lives, and changes in the dialysis method [17]. One study found that for people with CKD, adapting their routines to dialysis treatment allowed them to maintain their family relationships [44]. In this study we found that these normalization strategies also serve to bring continuity into a life that has been interrupted by CKD. Leading a normal life is more than just coping with the disease by controlling it [45].

Protective governing was originally defined as the tasks carried out by women with high-risk pregnancies to protect themselves and their babies from the threat of illness and death [52]. It places the emphasis on protecting oneself and someone else. This is relevant to CKD, since, as this study shows, along with other studies [53, 54], people who suffer from CKD take actions to protect both themselves and others.

There are abundant studies listed in the bibliography that deal with disease management and self-management as strategies employed by people with chronic diseases to promote their own health, manage the disease, and manage their lives with the disease [55–58] and thus normalize their lives [59]. Although this concept is useful when it is introduced into public policy to deal with chronic disease (for example Department of Health, 2008 [60]; Ministry of Health, Social Services and Equality–Government of Spain [61]), it leaves out the subjective aspect of the experience of living with a chronic disease; namely, taking care of oneself and others. In this study we focused on this aspect, adding a nuance to what is known about this experience.

Together with previous studies [28], this study has found that people mobilize to deal with the need for information and support that they do not find in institutions or from health professionals. Despite the increasing number of studies on what are termed *health movements*, this dimension is poorly documented in the experience of disease [49]. The present study does include it and presents it as a mechanism that supports the fulfillment of a normal life with a chronic renal disease.

## Conclusion

This study reveals that people with advanced CKD do not feel the same as before; they feel less, and so their actual real identity is continually called into question. Knowing what it feels like for people to live with a chronic disease is as important for clinicians and patients as knowing about symptoms and treatment.

Hence, the findings of this study contribute to an understanding of the experience of people with advanced CKD. It is hoped that it can help professionals in their relationships with CKD patients to respond to patients’ experiences and humanize current medical technologies. Given that in CKD health care professionals and specially nurses support patients in making decisions about their treatment [62], knowing what it means to live with advanced CKD will foster their empathy and help patients make these decisions. Hearing stories about the experience can help health care professionals guide wholistic, person-centered care. Specifically, through their intensive and long-term interaction with people in kidney replacement therapy, nurses working in renal units can develop relationships based on patients’ experiences.

The findings of this study speak of the resilience and adaptability of people with advanced CKD. The experience of chronic kidney disease is characterized by the effort to make life worth living. This should not go unnoticed by the health care professionals who care for these patients, so that they may support them effectively in their efforts to lead a normal life.

Finally, by describing the experience of people with advanced CKD, this study contributes to the design of instruments that measure quality of life with content validity; that is, with authenticity. It should not be forgotten that quality of life is a subjective concept, and that the meanings people give to their experiences must be included in these measurement tools.

## Supporting information

S1 File Field work matrix. (DOCX)
