# Supplementary material for: Living normally without being oneself: A qualitative study on the experience of living with advanced chronic kidney disease
Source: PLoS One. 2023 Dec 21;18(12):e0295506. doi: 10.1371/journal.pone.0295506 (PMC10734919; doi:10.1371/journal.pone.0295506)
Supplement: S1 File — (DOCX) [file pone.0295506.s001.docx]

**Field work matrix**

| **Participant/code** | **City** | **Position** | **Place** | **Product** |
| --- | --- | --- | --- | --- |
| Gabriela  **e1.Alc.271118.cf**  (1) | Alicante | Relative, daughter of sick person (died 1 year ago) | San Juan Beach cafe | FN  RI  TRANS |
| Alberto  **e2.Alc.160219.perc**  (2) | Alicante | Transplanted person with CKD | University Office | FN  RI  TRANS |
| Mario y Paula  **e3.Alc.190219.amb**  **(3)** | Alicante | Person with CKD, transplanted and who had graft rejection. Currently in HD.  Wife of person with CKD | University Office | FN  RI  TRANS |
| **María, Mariana**  **e4.Elch.230219.amb**  **(4)** | Elche | Person with CKD transplant, daughter with the same diagnosis and their husband and father | Participants' House | FN  RI  TRANS |
| **Liliana, Norma y Don Miguel**  **e5.Alp.270219.amb**  **(5)** | Alpatró | Person with CKD and mother and father of Isabel | Participants' House | FN  RI  TRANS |
| **Carmen**  **e6.Petrer.120319.perc**  **(6)** | Petrer | Person with CKD | Village cafe | FN  RI  TRANS |
| **Mónica y Mariana**  **e7.Elch020419.amb**  **(7)** | Elche | Person with CKD and their mother who is their caregiver | Participants' House | FN  RI  TRANS |
| **Sra. Marisol**  **e8.Alc.030419.cf**  **(8)** | Alicante | Caregiver wife of a retired person with CKD | Cafe near the Participants' House | FN  RI  TRANS |
| **Juan**  **e9.Alc.080419.perc**  **(9)** | Alicante | Person with CKD | University Office | FN  RI  TRANS |
| **Rocio**  **e10.Mur.180619.perc**  **(10)** | Murcia | Person with CKD | Association office | FN  RI  TRANS |
| **Mateo**  **e11.Mur.180619.perc**  **(11)** | Murcia | Person with CKD | Association office | FN  RI  TRANS |
| **Maribel and Carmen**  **(12)**  **e12.Mur.190619.amb** | Murcia | Person with CKD on hemodialysis and daughter who is a caregiver | Association office | FN  RI  TRANS |
| **Mario**  **e13.Ben.150719.perc**  **(13)** | Benidorm | Person with CKD on hemodialysis | Hemodialysis unit of the Hospital | FN  RI  TRANS |
| **Esperanza**  **e14.Ben.150719.perc.fc**  **(14)** | Benidorm | Person with CKD on hemodialysis | Hemodialysis unit of the Hospital | FN  RI  TRANS |
| **Victor**  **e15.Ben.150719.perc**  **(15)** | Benidorm | Person with CKD on hemodialysis | Hemodialysis unit of the Hospital | FN  RI  TRANS |
| **Alberta**  **e16.Ben.150719.cf**  **(16)** | Benidorm | Wife of person with CKD | Waiting room Hemodialysis unit of the Hospital | FN  RI  TRANS |
| **Cindy**  **e17.Ben.180719.perc**  **(17)** | Benidorm | Person with CKD on hemodialysis | Waiting room Hemodialysis unit of the Hospital | FN  RI  TRANS |
| **Carlos**  **e18.Ben.180719.perc**  **(18)** | Benidorm | Person with CKD on hemodialysis | Hemodialysis unit of the Hospital | FN  RI  TRANS |
| **Adalberto**  **e19.Ben.180719.perc**  **(19)** | Benidorm | Person with CKD on hemodialysis | Hemodialysis unit of the Hospital | FN  RI  TRANS |
| **Juanita**  **e20.Ben.180719.perc**  **(20)** | Benidorm | Person with CKD on hemodialysis | Hemodialysis unit of the Hospital | FN  RI  TRANS |
| **Milagros**  **e21.Ben.240719.perc**  **(21)** | Murcia | Civil association leader | Hemodialysis unit of the Hospital | FN  RI  TRANS |
| **Antonio**  **e22.Ben.290719.perc**  **(22)** | Benidorm | Person with CKD on hemodialysis | Hemodialysis unit of the Hospital | FN  RI  TRANS |
| **Maricruz**  **e23.Ben.290719.perc**  **(23)** | Benidorm | Person with CKD on hemodialysis | Hemodialysis unit of the Hospital | FN  RI  TRANS |
| **Raúl**  **(24)** | Alicante | civil association leader | Hemodialysis unit of the Hospital | FN  RI  TRANS |

Total interviews: **24**

Total participants interviewed:

**21 participants suffering from CKD**

**11 family caregivers**

Pseudonyms were used

FN: Field note

RI: Recording interviewers

Trans: Transcription
